# Clinical implications of insulin-like growth factor 1 system in early-stage cervical cancer

**DOI:** 10.1038/sj.bjc.6604661

**Published:** 2008-09-09

**Authors:** Y-F Huang, M-R Shen, K-F Hsu, Y-M Cheng, C-Y Chou

**Affiliations:** 1 Department of Obstetrics & Gynecology, College of Medicine, National Cheng Kung University, Tainan, Taiwan; 2 Department of Pharmacology, National Cheng Kung University, Tainan, Taiwan; 3 Center for Gene Regulation and Signal Transduction Research, National Cheng Kung University, Tainan, Taiwan

**Keywords:** insulin-like growth factor-1, insulin-like growth factor-1 receptor, IGF binding protein 3, cervical cancer, cervical carcinoma

## Abstract

This study was aimed to identify the expression and the correlation of insulin-like growth factor-1 (IGF-1) system and their prognostic impacts in cervical cancer. Seventy-two patients with early-stage cervical cancer were eligible. We obtained the serum levels of total IGF-1 and IGF binding protein-3 (IGFBP-3) by enzyme-linked immunosorbent assay and the expression of IGF-1 receptor (IGF-1R) in cancerous tissue by immuno-fluorescent (IF) stains. The 5-year recurrence-free and overall survival rates were significantly lower (*P*=0.003 and *P*=0.01, respectively) among patients with high-grade expression of tissue IGF-1R, compared with those with low-grade expression. After adjustment for other factors, preoperative serum total IGF-1 or IGFBP-3 levels failed to predict cancer death and recurrence. High-grade expression of IGF-1R and elevated preoperative squamous cell carcinoma antigen level were independent predictors of both death and recurrence, and combination of both factors could further help identify the subgroup of patients at higher death risk. The IF staining indicates the colocalisation of IGF-1 and IGF-1R in the cancerous tissues, whereas the IGF-1R expression is not correlated with circulating levels of IGF-1 or IGFBP-3. In early-stage cervical cancer, IGF-1 system may have a paracrine or autocrine function and the adverse impacts on prognosis by IGF-1R overexpression are implicated.

Cancer of the cervix is the second most common cancer among women worldwide (Schoell *et al*, 1999). An estimated 500 000 cases are newly diagnosed and nearly 200 000 deaths are attributable to the disease in the world annually. A growing body of evidence has accumulated to indicate that oncogenic types of human papillomavirus (HPV) serve as an important factor in the development of the precursors of cervical cancer (zur Hausen, 1991; Syrjänen *et al*, 1992; Muñoz *et al*, 2003). The protein products of HPV DNA appear to be interactive with the antioncogenic function of the retinoblastoma gene and the p53 gene. However, only a small fraction of those infected by HPV develop cancer, indicating that other factors contribute to the progression to cervical cancer. Despite intensive investigation, the tumour biology of this disease is still largely unknown. Although prognostic factors such as pelvic lymph node metastasis affects the outcome of cervical cancer, the variability in progression-free and overall survival (OS) among patients with similar clinical and pathological characteristics makes it difficult to predict the outcome reliably.

The insulin-like growth factor 1 (IGF-1) system is comprised of ligand (IGF-1), receptor (IGF-1R) and a family of binding proteins (IGF-BPs). The insulin-like growth factor 1 is a 7.6 kDa polypeptide secreted in the liver and is also synthesized widely in other tissues (Jones and Clemmons, 1995). In addition to endocrine function, potential paracrine and autocrine functions are also revealed in IGF-responsive tissues, such as epiphyseal growth plate, ovary and uterus. Insulin-like growth factor binding protein 3 (IGFBP-3), a 29–48 kDa protein that localises outside the cell surface, is the major serum carrier of IGF to IGF-1R. Most actions of IGF-1 are mediated through IGF-1R signalling, resulting in mitogenic and metabolic functions *in vivo* as well as *in vitro* (Yu and Rohan, 2000). The binding of IGF-1R is suppressed by IGFBPs, especially IGFBP-3, producing an inhibitory effect on cell growth. There is a controversy over the complex feature of IGF-1 system in carcinogenesis. In a systemic review, high serum IGF-1 levels were associated with increased risk of prostate and premenopausal breast cancer, but not with increased risk of lung cancer or colorectal cancer (Renehan *et al*, 2004). As to IGFBP-3, high concentrations were only correlated to increased risk of premenopausal breast cancer. On the other hand, one study showed that IGFBP-3 may have a protective effect in ovarian cancer (Dal Maso *et al*, 2004).

Earlier we have investigated the regulatory mechanism of IGF-1R signalling and its importance in cervical cancer formation by cell lines and animal model (Shen *et al*, 2006). The growth and invasiveness of cervical cancer cells were dose-dependently stimulated by IGF-1, whereas those of normal cervical epithelial cells were not. The treatment of blocking antibody of IGF-1R remarkably decreased IGF-1R phosphorylation and downstream activation of Akt and Erk1/2, hereby inhibiting tumour growth and causing tumour regression in SCID mice model. Little information is available on the clinical implication of IGF-1 system in cervical cancer except for a small clinical study showing higher serum level of IGF-1 in women with squamous intraepithelial lesion than normal control (Wu *et al*, 2003). This study aims to investigate the association of IGF-1 system and clinical outcome of cervical cancer, by analysing the serum levels of IGF-1 and IGFBP-3, and tissue abundances of IGF-1R for the same patient. The results suggest the important function of IGF-1 system in predicting the clinical outcome of patients with early-stage cervical cancer.

## Materials and methods

### Patient population

Patients were identified through the Gynaecology Service database at National Cheng Kung University Hospital, Taiwan, between January 2000 and November 2002. They have been followed up regularly, and the date of latest record retrieved was December 31, 2007. We included patients with early-stage carcinoma of uterine cervix who experienced radical or modified radical hysterectomy and pelvic lymphadenectomy. The staging met the criteria of the clinical staging of International Federation of Gynaecology and Obstetrics (FIGO). The histological classification was defined according to the World Health Organization classification. The cervical tumour volume of surgical specimen was calculated using the formula: *π*/6 × (R_1_ × R_2_ × R_3_) (Chou *et al*, 1994). Preoperative serum levels of tumour markers were available. Initially, 263 patients of newly diagnosed cervical cancer between 2000 and 2002 were recruited into our study. However, patients who had minimal residual tumours, who had experienced hysterectomy elsewhere or who received primary therapy instead of surgery were excluded: 40 had undergone conization, 28 with stage IA disease, 11 were referred for postoperative treatment, and 75 preferred irradiation or concurrent chemoradiation as the primary therapeutic modality. Besides, informed consents were not available in another 37 patients. Finally, 72 participants were enroled for this study and for final analysis. The research protocol and consent form were approved by the institutional review board. We reviewed the medical records and pathological slides, which provided clinical characteristics, pathological diagnosis, treatment and outcome information.

### Clinical definitions

Both OS and recurrence-free survival (RFS) were calculated from the date of diagnosis. The duration of OS was measured to the date of death from any cause; survivors were censored on the date they were last known to be alive. The duration of RFS was designated to the first clinical recurrence or death from any cause, unless the patient was recurrence-free at the time of last contact, in which case it was measured to the date of last contact. The preoperative body mass index (BMI) was calculated using the universal formula: the weight/the square of the height (kg/m^2^).

### Levels of total IGF-I and IGFBP-3 in preoperative serum

A non-extraction IGF-1 enzyme-linked immunosorbent assay (ELISA) kit (DSL-10-2800) and IGFBP-3 ELISA kit (DSL-10-6600) from Diagnostics Systems Laboratories, Inc., Webster, TX, USA was used for the determination of preoperative serum IGF-1 and IGFBP-3 levels. Samples were tested in duplicates and repeated if the correlation coefficient between the absorbance and the amount in the standards was less than 0.95.

### Immunofluorescent stains of cervical cancer tissue samples

Those surgical specimens were processed for immunofluorescent (IF) stains using monoclonal antibodies against IGF-1 (clone M23, Catalogue Number CBL52, Chemicon International, Temecula, CA, USA) or IGF-1R (clone 24–31, Catalogue Number MAB1120, Chemicon International). The monoclonal antibody against IGF-1R recognises the *α*-subunit of IGF-1R with the epitope between amino acid 283–440 (exon 4–6) of IGF-1R and shows no cross-reaction with insulin receptor (Schumacher *et al*, 1993; Yu and Yoko, 2007). We used Alexa 488-labeled or Alexa 594-labeled secondary antibodies (Molecular Probes, OR) and Hoechst 33258 (Sigma-Aldrich, Dorset, UK) for immunofluorescence. The IF stains were calculated manually using a CoolSnap-Pro colour digital camera (Roper Scientific, Trenton, NJ, USA) in 15–20 high-power fields. The intensity and distribution of IF stains of IGF-1R were graded using a scale of 1–4 in each specimen. Grade 1 indicates that IGF-1R staining intensity and distribution are less than 25% of tumour area. Grade 2 indicates that the staining intensity and distribution are more than 25%, but less than 50% of tumour area. Grade 3 indicates that the staining intensity and distribution are more than 50%, but less than 75% of tumour area. Grade 4 indicates that the staining intensity and distribution are more than 75% of tumour area. Human placenta was used as the positive control for IGF-1R and IGF-1. A negative control, for which the primary antibody was substituted with the same concentration of the appropriate immunoglobulin G, was used in each staining run. Sections of each patient's specimen were examined for histology, intensity and distribution of IF stains of IGF-1 and IGF-1R by two trained investigators who were blinded to each patient's clinical history.

### Statistics

Data were analysed by SPSS (version 13.0; SPSS Inc, Chicago, IL, USA). The possible prognostic factors included age (>51 *vs* ⩽51), FIGO stage (⩾IIA, IB2 *vs* ⩽IB1), cell type (adenocarcinoma, others *vs* squamous cell carcinoma), tumour volume (>11.38 *vs* ⩽11.38 cm^3^), preoperative squamous cell carcinoma antigen (SCC Ag) level (>2.5 *vs* ⩽2.5 ng ml^−1^), carcinoembryonic antigen (CEA) level (>3.5 *vs* ⩽3.5 ng ml^−1^), parametrial invasion (yes *vs* no), pelvic lymph node metastases (yes *vs* no), BMI (>25 *vs* ⩽25), adjuvant therapy (radiotherapy (RT) alone, chemotherapy (CT) alone, RT+CT *vs* no), tissue IGF-IR overexpression (high *vs* low), serum total IGF-1 level (⩽125.11 *vs* >125.11 ng ml^−1^) and serum IGFBP-3 level (⩽3499.82 *vs* >3499.82 ng ml^−1^). The cutoff points were mainly chosen based on Receiver Operating Curve analysis. Survival data were assessed by Kaplan–Meier method. Univariate analysis by the log-rank test evaluates the possible influence on survival or disease recurrence covariates. Multivariate Cox proportional hazards model by stepwise methods was performed to test the independent function of each screened prognostic variables of univariate analysis. Student's *t*-test for continuous variables was used to test the difference between two subgroups of serum IGF-1 or IGFBP-3 levels, which were categorized by the other categorical variables. The correlation between serum IGF-1/IGFBP-3 and other continuous parameters was analysed with linear regression model. All *P*-values presented are two-sided and considered as statistically significant when *P*⩽0.05.

## Results

### Patterns of intratumour IGF-1 and IGF-1R expression in cervical cancer

**Figure 1:**
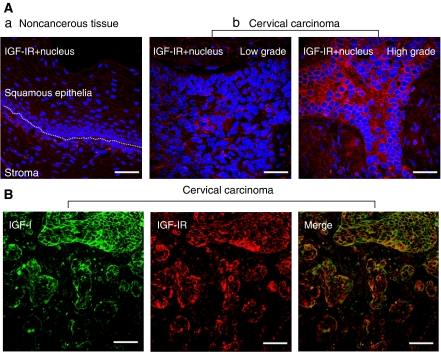
(**A**) Pattern of IGF-1R expression in the surgical specimens of cervical cancer. (a) IGF-1R protein was scanty in normal or noncancerous cervical tissues of all surgical specimens examined (*n*=72). (b) Adjacent cervical cancerous tissues clearly expressed IGF-1R protein with different abundances. Representative pictures for cervical cancer samples with different IGF-1R grades. Low grade indicates that the distribution of IGF-1R staining is less than 50% of tumour area, whereas high grade indicates that the distribution of IGF-1R staining is more than 50% of tumour area. The double-staining technique was used to identify IGF-1R (red) and nucleus (blue) in cervical cancer tissue. Nuclei were stained with Hoechst 33258. Scale bar, 30 *μ*m. (**B**) Colocalisation of IGF-1 and IGF-1R in cervical cancer tissues. The double-staining technique was used to identify IGF (green) and IGF-1R (red) in cervical cancer tissues. A representative picture of samples of cervical cancer. Scale bar, 40 *μ*m.

We at first examined the expression level of IGF-1R in surgical specimens of early-stage cervical cancer by IF staining. As shown in Figure 1Aa, IGF-1R protein was scanty in normal or noncancerous cervical tissues of all surgical specimens examined (*n*=72). In contrast, cervical cancer tissues clearly expressed IGF-1R protein with different amounts (Figure 1Ab). In addition, the double stainings of IGF-1R and IGF-1 indicate the colocalisation of IGF-1 and IGF-1R in the cancerous tissues (Figure 1B).

### Clinicopathological characteristics and clinical outcome

The patient characteristics were shown in Table 1. We first analyse the patient characteristics to identify the possible predictors by the univariate analysis (Table 1). Of the 10 covariates, 7 were related to increased relative risk of death and disease recurrence, including FIGO staging, tumour size, preoperative SCC Ag and CEA levels, parametrial invasion, lymph node metastasis and adjuvant therapy. These seven potential prognostic factors were included subsequently in multivariate analysis with IGF-1 system.

### The correlation between serum IGF-1/IGFBP-3 and other prognostic factors

Further, we analysed the relationship between serum IGF-1/IGFBP-3 and other variables. By linear regression model, we found IGF-1 levels may be related to preoperative CEA levels and tumour volumes with marginal statistical significance (*P*=0.07 and *P*=0.08, respectively). In addition, there may be certain association between IGFBP-3 and lymph node metastasis (*P*=0.05), and BMI (*P*=0.06).

### IGF-1 system and clinical outcome

#### Univariate association between IGF-1 system and clinical outcome

**Figure 2:**
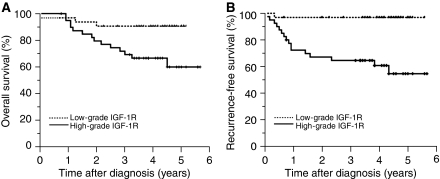
Kaplan–Meier estimates of overall survival and recurrence-free survival by IGF-1R overexpression in cervical cancer specimens. (**A**) Five-year overall survival rates are 90.6 and 59.9% in patients with low-grade and high-grade IGF-1R overexpression, respectively (*P*=0.01). (**B**) Five-year recurrence-free survival rates are 96.9 and 54.6% in patients with low-grade and high-grade IGF-1R overexpression, respectively (*P*=0.003).

We grouped the patients by IGF-1R grades, serum levels of IGF-1 or IGFBP-3 and analysed the clinical outcome accordingly (Table 2). The patients were further categorized as low-grade (grade 1–2) and high-grade IGF-1R expressions (grade 3–4) (Figure 1Ab) because of the limited case numbers in each category. Of the 72 patients with cervical cancer assessed, 17 deaths and 17 recurrences were encountered. In the univariate analysis, the tissue expression level of IGF-1R is a significant predictor of death as well as disease recurrence. The 5-year RFS and OS rates were 96.9 and 90.6% among patients with low-grade overexpression of tissue IGF-1R, whereas RFS and OS were 54.6 and 59.9% among those with high-grade overexpression (Figure 2). The serum level of IGF-1 is a significant predictor of survival (relative risk, 4.17 (95% CI: 1.20–14.55)), but not disease recurrence. However, the circulating level of IGFBP-3 is not associated with risk of survival and disease recurrence.

#### Multivariate association between IGF-1 system and clinical outcome

**Figure 3:**
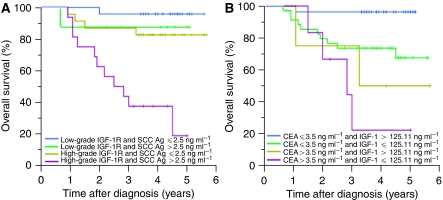
Kaplan–Meier curves for overall survival in patient subgroups. (**A**) Survival differences in 72 patients with low or high IGF-1R expression and SCC Ag less than or greater than 2.5 ng ml^−1^ were significant (*P*=0.001). (**B**) Survival differences in patients with CEA less than or greater than 3.5 ng ml^−1^ and serum IGF-1 greater than or less than or 125.11 ng ml^−1^ were significant (*P*<0.001).

By multivariate Cox regression analysis, high-grade overexpression of IGF-1R alone and elevated preoperative SCC Ag level alone were both independent predictors of death and recurrence (Table 3). Assuming proportional hazards, more than 80% power was estimated when testing the hypothesis of increased risk in OS and RFS among those with high-grade expression of IGF-1R relative to that among those with low-grade expression. Elevated preoperative CEA level and stage ⩾IIA were of significant predictive value of death and recurrence, respectively. After adjustment for other factors, preoperative serum total IGF-1 or IGFBP-3 levels failed to predict cancer death and recurrence. By two-covariate prognostication, combination of increased SCC Ag level and high-grade IGF-1R overexpression or combination of elevated CEA level and lower IGF-1 level were remarkably related to advancing death risk. The 5-year OS were illustrated in Figure 3. Moreover, the serum level of IGF-1 or IGFBP-3 did not statistically differ between the two distinct IGF-1R overexpressions (*P*=0.77 and *P*=0.07, respectively), indicating no direct correlation between circulating IGF-1 or IGFBP-3, and IGF-1R overexpression in cervical cancer cells.

## Discussion

This study is a retrospective analysis of patients with early-stage cervical cancer who received surgery as their primary treatment. We provide evidence that IGF-1 system has a certain function in tumour formation and clinical outcome of cervical cancer. This conclusion is supported by the following findings: (i) high-grade overexpression of IGF-1R is an independent predictor of cervical cancer death and recurrence, and when combined with elevated serum SCC Ag level could further help identify the subgroup of patients at higher death risk. (ii) Preoperative serum total IGF-1 or IGFBP-3 levels failed to predict cervical cancer death and recurrence. (iii) The colocalisation of IGF-1 and IGF-1R in the cancerous tissues, and the lack of correlation between circulating IGF-1 or IGFBP-3, and IGF-1R overexpression in cervical cancer cells suggest likely autocrine or paracrine IGF-1 stimulation of IGF-1R signalling.

IGF-1R has been believed to be pivotal in tumorigenesis. Defective IGF-1R has been shown to inhibit cellular transformation and tumorigenesis (Prager *et al*, 1994). Complexity in IGF signalling pathways has not been well established in cervical cancer. Steller *et al* described that overexpression of IGF-1R in cervical cancer cell lines and explants controlled the proliferation of cancer cells (Steller *et al*, 1996). We have pointed out that IGF-1, as a stimulator through IGF-1R signalling, interacted with *α*_v_*β*_3_ integrin in cervical cancer cell invasiveness and proliferation (Shen *et al*, 2006). Further, we recognised that IGF-1 stimulated potassium-chloride cotransporters, which were required for invasion and proliferation of cervical cancer, ovarian cancer and breast cancer cells (Shen *et al*, 2004; Hsu *et al*, 2007). Related pathways on the synthesis, expression, ligand-receptor interaction and tyrosine-kinase activation of IGF-1R in cervical cancer deserve further clarifications.

The function of IGF-1R expression in disease outcome in cervical cancer has rarely been discussed in earlier literature. Our present study reported the significantly adverse impacts on RFS and OS in patients with high-grade IGF-1R overexpression. Therefore, the application of IGF-1R overexpression may be rational to predict clinical prognosis in operable cervical cancer.

A reliable serum biomarker to predict occurrence, progression or prognosis of human cancers can offer early diagnosis or surveillance of therapy efficacy. Scientists have contributed to investigating the correlation of serum IGF-1 or IGFBP-3 to the prevalence of a variety of cancers, but have given diverse results. Higher circulating IGF-1 level was associated with higher risk of prostate cancer (Chan *et al*, 1998), lung cancer (Yu *et al*, 1999), colorectal cancer (Ma *et al*, 1999), premenopausal breast cancer (Toniolo *et al*, 2000) and younger aged ovarian cancer (Peeters *et al*, 2007). However, in a systemic review (Renehan *et al*, 2004), the correlation was not significant in lung cancer and colorectal cancer after adjustment. As to IGFBP-3, higher serum IGFBP-3 levels to the increased risk of premenopausal breast cancer (Renehan *et al*, 2004) and distant recurrence of postmenopausal and estrogen receptor-positive breast cancer (Goodwin *et al*, 2002) were observed. In contrast, elevated IGFBP-3 levels may have a protective function in ovarian cancer occurrence (Dal Maso *et al*, 2004). As to cervical cancer, studies showed lower serum IGF-1 level correlated to increased risk of cervical cancer (Serrano *et al*, 2006) and its precancer lesions (Schaffer *et al*, 2007). One study inferred that the IGFBP-3 level could be used in predicting prognosis of cervical cancer (Mathur *et al*, 2003), but the study result should be limited because of small sample size and concealed stage. Our study illustrated that in early-stage cervical cancer patients, lower IGF-1 level seemed to be associated with worse OS but was not of an independent value, and there was no relationship between IGFBP-3 and survival. It is still unclear why increased serum IGF-1 level may have a sheltery effect on the risk of cervical cancer, whereas the unfavourable effect of serum IGF-1 is addressed in certain sex hormone-related cancers, such as prostate cancer or breast cancer. One of the theories is that the natural history to cervical cancer may differ from sex hormone-related cancers (Schaffer *et al*, 2007). Second, the different effects may be generated by the differences between Asian and Caucasian patients, related to socioeconomic and diet influences. In our study, serum levels of IGF-1 and IGFBP-3 did not differ between two subgroups of IGF-1R overexpression with colocalised IGF-1 in the specimen sections. The possible hypothesis is that the IGF system may contribute to the regional influences in the battlefield, suggesting paracrine or autocrine function in agreement on prior suppositions (Bergmann *et al*, 1995; Schips *et al*, 2004; Schillaci *et al*, 2005). More candidates should be enroled in further studies to realise the differences of serum levels of IGF-1 between normal individuals, patients with precancer lesions and cervical cancer.

Pretreatment SCC Ag or CEA assays, as tumour-associated antigens, have been identified to be valuable in distinguishing high-risk patients requiring adjuvant therapy and in predicting prognosis in early-stage cervical cancer (Bae *et al*, 1997; Takeda *et al*, 2002; Reesink-Peters *et al*, 2005). In our cohorts, the integration of SCC Ag level and IGF-1R overexpression or the combination of CEA and IGF-1 levels could produce more accurate estimates in identifying patient subgroups of poor prognosis.

On the basis of former results of IGF-1R expression of primary site or possible circulating tumour cells in many cancers, targeting therapy with IGF-1R antibody was enormously developed within recent years (Bradley and Douglas, 2005; Gotlieb *et al*, 2006; de Bono *et al*, 2007; Hartog *et al*, 2007; Imsumran *et al*, 2007). Although many issues, such as the potential blockade of endocrine, nervous and myocardial functions, or the application in conjugation with conventional chemotherapy, were raised, this new strategy may bring new hope in cancer treatment.

## Figures and Tables

**Table 1:** Patient characteristics and univariate analysis for relative risk of death and disease recurrence of clinicopathological factors

**Characteristics**	***n***	**%**	**RR of death**	***P***	**RR of recurrence**	***P***
*Age*	Median, 51					
	Range, 31–77					
⩽51	37	51.4	1		1	
>51	35	48.6	0.97	0.95	0.92	0.86
						
*FIGO stage*						
⩽IB1	56	77.8	1		1	
IB2	10	13.9	2.05	0.28	2.70	0.14
⩾IIA	6	8.3	8.37	<0.001	14.44	<0.001
						
*Cell type*						
Squamous cell carcinoma	53	73.6	1		1	
Adenocarcinoma	15	20.8	1.13	0.84	0.69	0.56
Others	4	5.6	<0.001	0.98	<0.001	0.98
						
*Tumor volume (cm^3^)*	Median, 6.65					
	Range, 0–82.47					
⩽11.38	45	62.5	1		1	
>11.38	27	37.5	7.21	0.001	11.05	<0.001
						
*Preoperative SCC Ag (ng ml^−1^)*	Median, 1.40					
	Range, 0–62.3					
⩽2.5	48	66.7	1		1	
>2.5	24	33.3	6.23	0.001	4.83	0.002
						
*Preoperative CEA (ng ml^−1^)*	Median, 1.10					
	Range, 0–13.7					
⩽3.5	62	86.1	1		1	
>3.5	10	13.9	3.92	0.007	3.65	0.01
						
*BMI*	Median, 24.76					
	Range, 16.30–33.12					
⩽25	38	52.8	1		1	
>25	31	43.1	0.77	0.60	1.50	0.42
						
*Parametrial involvement*						
No	52	72.2	1		1	
Yes	20	27.8	3.27	0.02	3.11	0.02
						
*Lymph node metastases*						
No	55	76.4	1		1	
Yes	17	23.6	3.36	0.01	4.37	0.003
						
*Adjuvant therapy*						
No	21	29.2	1		1	
RT alone	19	26.4	2.32	0.33	2.45	0.30
CT alone	16	22.2	1.45	0.71	0.68	0.76
RT+CT	16	22.2	7.56	0.01	10.66	0.002

BMI=body mass index; CEA=carcinoembryonic antigen; CT=Chemotherapy; FIGO=Federation of Gynecology and Obstetrics; RR=relative risk; RT=Radiotherapy; SCC Ag=squamous cell carcinoma antigen.

**Table 2:** Univariate analysis for relative risk of death and disease recurrence of IGF-1 system

**Variable**	***n***	**%**	**RR of death**	***P***	**RR of recurrence**	***P***
*IGF-1R expression*						
1	22	30.6	—		—	
2	10	13.8	—		—	
3	28	38.9	—		—	
4	12	16.7	—		—	
Low-grade (grade 1–2)	32	44.4	1		1	
High-grade (grade 3–4)	40	55.6	4.31	0.02	15.95	0.007
						
*Serum total IGF-1 (ng ml^−1^)*	Median, 119.22					
	Range, 9.11–330.21					
>125.11	31	43.1	1		1	
⩽125.11	41	56.9	4.17	0.03	2.00	0.20
						
*Serum IGF-BP3 (ng ml^−1^)*	Median, 3322.91					
	Range, 1564.00–6529.00					
>3499.82	28	38.9	1		1	
⩽3499.82	44	61.1	1.63	0.36	1.26	0.65

IGF-BP3=IGF binding protein-3; IGF-1=insulin-like growth factor-1; IGF-1R=IGF-1 receptor; RR=relative risk.

**Table 3:** Multivariate analysis for relative risk of death and disease recurrence of prognostic factors by the Cox proportional hazard model

**Variable**	**RR of death**	**95% CI**	***P***
Preoperative SCC Ag (>2.5 *vs* ⩽2.5 ng ml^−1^)	3.49	1.11–10.99	0.03
Preoperative CEA (>3.5 *vs* ⩽3.5 ng ml^−1^)	3.01	1.09–8.31	0.03
IGF-1R expression (high *vs* low)	5.66	1.26–25.47	0.02
Serum total IGF-1 level (⩽125.11*vs* >125.11 ng ml^−1^)	2.90	0.76–11.13	0.12
			
	**RR of recurrence**	**95% CI**	***P***
FIGO stage (⩾IIA *vs* ⩽IB1)	7.61	2.37–22.78	0.001
Preoperative SCC Ag (>2.5 *vs* ⩽2.5 ng ml^−1^)	4.39	1.45–13.26	0.009
IGF-1R expression (high *vs* low)	10.06	1.25–81.30	0.03
Serum total IGF-1 level (⩽125.11*vs* >125.11 ng ml^−1^)	1.30	0.40–4.23	0.67

CEA=carcinoembryonic antigen; CI=confidence interval; FIGO=Federation of Gynecology and Obstetrics; IGF-1=insulin-like growth factor-1; IGF-1R=IGF-1 receptor; RR=relative risk; SCC Ag=squamous cell carcinoma antigen.
